# Conjoined Twins in a Triplet Pregnancy: A Case Report

**DOI:** 10.1155/2011/235873

**Published:** 2011-09-06

**Authors:** Lynn J. Shepherd, Graeme N. Smith

**Affiliations:** Department of Obstetrics & Gynaecology, Kingston General Hospital, Queen's University, Victory 4, 76 Stuart Street, Kingston, ON, Canada K7L 2V7

## Abstract

*Background*. Conjoined twins are a rare complication of monozygotic twinning and are associated with high perinatal mortality. *Case*. Here we present a case of conjoined twins in a triplet pregnancy diagnosed at 13 of weeks gestation. With the aid of 3D ultrasound and MRI images, the parents were counseled regarding the management options, including continuation of pregnancy, termination of pregnancy, or selective fetocide. They chose selective fetocide of the conjoined twins and went on to deliver the remaining triplet at term. *Conclusion*. This case represents to our knowledge the only MRI images of conjoined twins in a triplet pregnancy and demonstrates how 3D imaging can be used to better counsel patients about management options.

## 1. Introduction

Conjoined twins occur when division of a single zygote takes place twelve to thirteen days after fertilization [1]. Fetuses share not only a single amniotic cavity but also parts of their bodies. The incidence is estimated at 1 in 80,000 deliveries [2]. Conjoined twins within a triplet pregnancy are very rare and a recent review suggested the incidence is less than one in a million deliveries [3]. The purpose of this report is to describe the presentation and management of a dichorionic triplet pregnancy complicated by conjoined twins.

## 2. Case Report

A thirty-two-year-old gravida 1 para 0 was referred to High Risk Obstetrics with a diagnosis of dichorionic diamniotic twin gestation. She had been treated with clomiphene citrate to conceive following a history of unexplained infertility. An early ultrasound at six weeks gestation was reported as a dichorionic diamniotic twin gestation. This was based on a crown rump length (CRL) of 10.1 mm for fetus A and 16.8 mm for fetus B. Two separate and distinct fetal heartbeats were also reported. 

**Figure 1:**
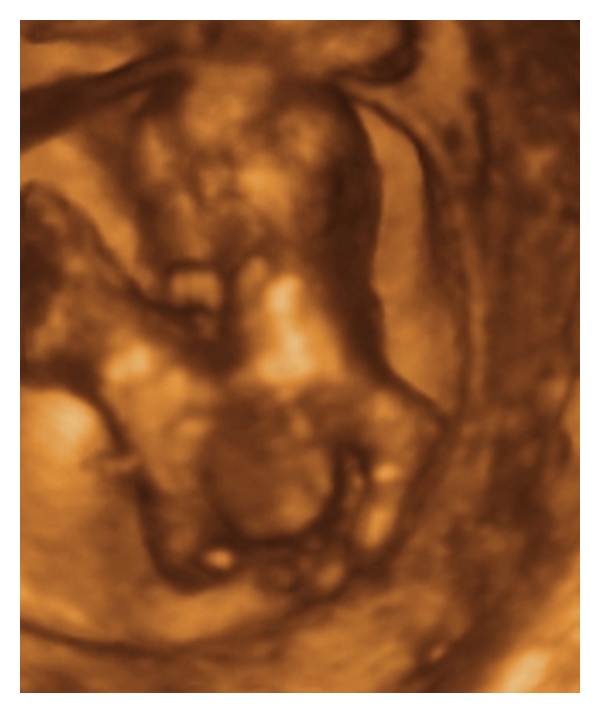
3D image of conjoined twins.

**Figure 2:**
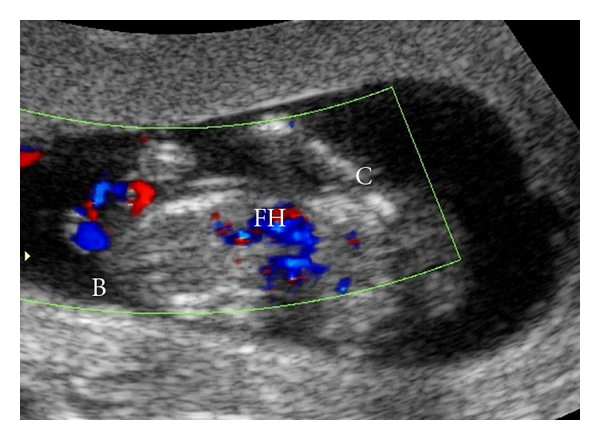
Shared fetal heart of conjoined twins.

**Figure 3:**
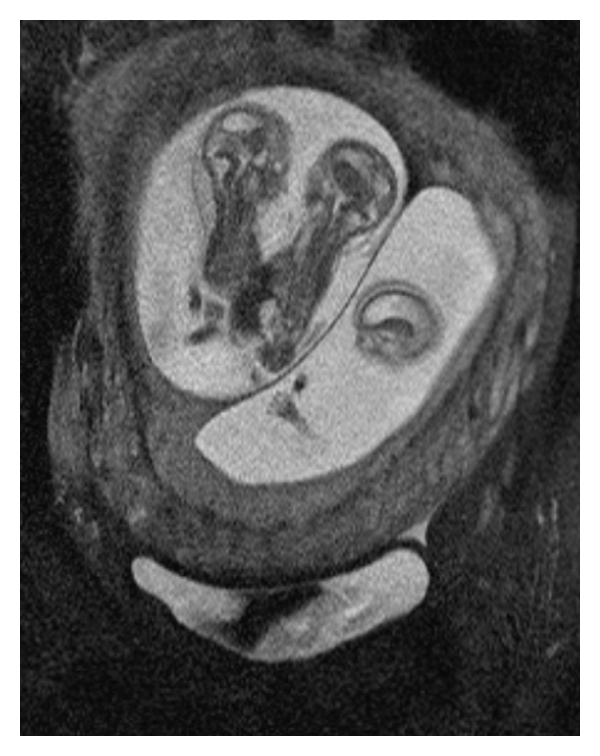
MRI images of conjoined twins in a triplet pregnancy.

The patient wished to participate in Integrated Prenatal Screening, and she presented for a nuchal translucency at thirteen weeks and one day of gestation. This scan identified a triplet gestation complicated by a set of conjoined twins. One amniotic sac contained a single fetus (triplet A) with a CRL of 74 mm and a nuchal translucency of 1.5 mm. Within the second amniotic sac, triplets B and C, were joined through the thorax and abdomen, with four arms, four legs and a single heart centrally located between the two fetuses. The CRL for triplet B was 62.2 mm, and the nuchal translucency was measured at 3.9 mm. The CRL for triplet C was 62.5 mm, and a cystic hygroma was also seen. The nuchal translucency for Triplet C was 7.1 mm. Using 3D ultrasound and MRI, a diagnosis of thoracoomphalopagus was confirmed (see Figures 1–3).

The parents were counseled and options discussed included (1) continue the pregnancy understanding that the shared heart meant the conjoined twins could not be separated should they survive to delivery, (2) terminate the entire pregnancy or, (3) selective feticide of the conjoined twins. After further counseling and deliberation the parents chose the third option. This was carried out at 13 weeks and five of days gestation by intracardiac potassium chloride injection. A repeat scan was performed four days later again confirming a viable singleton gestation with fetal demise of conjoined twins. The patient went on to have another scan at 17 weeks and 6 days of gestation, which showed normal anatomy and interval growth of the remaining triplet. The nonviable conjoined twins were noted at the time of that scan. 

The pregnancy continued without complication, and a healthy male infant was delivered by low forceps for maternal exhaustion weighing 3590 grams at 40 of weeks gestation. Apgars were 7 and 9. The vestiges of the conjoined twins were not visible at the time of delivery. The mother and infant were discharged home on postpartum day four.

## 3. Discussion

A review published in 2003 reported thirteen cases of conjoined twins within a triplet pregnancy [2]. Ultrasound was used to make the diagnosis in the first trimester in six cases (46%) [2, 4–7], in the second trimester in six cases (46%) [8–14], and in the third trimester in the remaining case (8%) [15]. Four of the patients chose to terminate the entire pregnancy (31%). Three patients chose expectant management. One of these women was delivered by elective section at 36 weeks with all three infants surviving. The remaining two were delivered prior to 32 weeks following early rupture of membranes and preterm labour. Five patients had a selective termination of the conjoined twins. Three of these women went on to deliver at term without complication. All three pregnancies were dichorionic. The remaining two selective reductions resulted in intrauterine fetal demise of the nonconjoined triplets; one at the time of intracardiac injection and the other at 28 weeks following cord entanglement. Both of these pregnancies were monochorionic. The final case reported no intervention but subsequent early intrauterine demise of the conjoined twins.

In the review [2], three of the thirteen pregnancies were conceived using artificial reproduction technology (ART). Recent data suggests the rate of monozygotic twinning is increased with ART, specifically when blastocysts are transferred [16–18]. It is thought that manipulation of the zona pellucida, as in assisted hatching [19, 20] and intracytoplasmic sperm injection (ICSI) [21], further increases the risk of monozygotic twins and by extension of conjoined twins. A review of the literature reveals nine published cases of conjoined twins, triplet or quadruplet pregnancies conceived using ART [22]. Seven of the nine cases used assisted hatching or ICSI. 

A false positive diagnosis of conjoined twins has been reported [23] identifying the need for further imaging modalities to confirm the diagnosis and facilitate counseling of the woman and her partner. There is no evidence that MRI is associated with any risk to the developing fetus [24].

## 4. Conclusion

To our knowledge, this case represents the first MRI images of conjoined twins in a triplet pregnancy. This case was diagnosed at 13 weeks using conventional ultrasound. Further evaluation with 3D ultrasound and MRI was used to provide images used in counseling the parents (see Figures 1 and 2). The quality and clarity of the 3D ultrasound and MRI images allowed the parents to fully understand the extent and severity of the deformity which enabled them to reach an informed decision on management.
